# Propionibacteria as promising tools for the production of pro-bioactive scotta: a proof-of-concept study

**DOI:** 10.3389/fmicb.2023.1223741

**Published:** 2023-07-31

**Authors:** Roberta Coronas, Giacomo Zara, Antonio Gallo, Gabriele Rocchetti, Marco Lapris, Giacomo Luigi Petretto, Severino Zara, Francesco Fancello, Ilaria Mannazzu

**Affiliations:** ^1^Department of Agricultural Sciences, University of Sassari, Sassari, Italy; ^2^Department of Animal Science, Food and Nutrition (DIANA), Faculty of Agricultural, Food and Environmental Sciences, Università Cattolica del Sacro Cuore, Piacenza, Italy; ^3^Department of Medicine, Surgery and Pharmacy, University of Sassari, Sassari, Italy

**Keywords:** pro-bioactive scotta, propionibacteria, by-products valorization, vitamin B12, vitamin B9, circular economy

## Abstract

Dairy propionibacteria are Gram positive Actinomycetota, routinely utilized as starters in Swiss type cheese making and highly appreciated for their probiotic properties and health promoting effects. In this work, within the frame of a circular economy approach, 47 *Propionibacterium* and *Acidipropionibacterium* spp. were isolated from goat cheese and milk, and ewe rumen liquor, and characterized in view of their possible utilization for the production of novel pro-bioactive food and feed on scotta, a lactose rich substrate and one of the main by-products of the dairy industry. The evaluation of the Minimum Inhibitory Concentration (MIC) of 13 among the most common antibiotics in clinical practice revealed a general susceptibility to ampicillin, gentamycin, streptomycin, vancomycin, chloramphenicol, and clindamycin while confirming a lower susceptibility to aminoglycosides and ciprofloxacin. Twenty-five isolates, that proved capable of lactose utilization as the sole carbon source, were then characterized for functional and biotechnological properties. Four of them, ascribed to *Propionibacterium freudenreichii* species, and harboring resistance to bile salts (growth at 0.7–1.56 mM of unconjugated bile salts), acid stress (>80% survival after 1 h at pH 2), osmostress (growth at up to 6.5% NaCl) and lyophilization (survival rate > 80%), were selected and inoculated in scotta. On this substrate the four isolates reached cell densities ranging from 8.11 ± 0.14 to 9.45 ± 0.06 Log CFU mL^−1^ and proved capable of producing different vitamin B9 vitamers after 72 h incubation at 30°C. In addition, the semi-quantitative analysis following the metabolomics profiling revealed a total production of cobalamin derivatives (vitamin B12) in the range 0.49–1.31 mg L^−1^, thus suggesting a full activity of the corresponding biosynthetic pathways, likely involving a complex interplay between folate cycle and methylation cycle required in vitamin B12 biosynthesis. These isolates appear interesting candidates for further *ad-hoc* investigation regarding the production of pro-bioactive scotta.

## 1. Introduction

Due to the increasing awareness of consumers on the importance of a healthy diet, both the scientific community and the agri-food industry are being engaged in the development of high-quality novel foods that, by delivering prebiotics, probiotics, microbial metabolites, and bioactive compounds, may combine synbiotic (prebiotic plus probiotic) or pro-bioactive (probiotic plus bioactive compounds) properties (Cunningham et al., 2021). Since fermented foods are the best candidates to reach such objective (Beltrán-Barrientos et al., 2016; Revuelta et al., 2018; Rocchetti et al., 2019; Tiss et al., 2020; Diez-Ozaeta and Astiazaran, 2022), the screening of microbes with suitable biotechnological and functional properties is required. Among the possible microbial candidates, propionibacteria seem to fit the purpose. The family *Propionibacteriaceae* includes the genera *Propionibacterium* spp., *Acidipropionibacterium* spp., *Pseudopropionibacterium* spp., and *Cutibacterium* spp. (Scholz and Kilian, 2016). Strains of *P. freudenreichii* and *A. acidipropionici* (formerly *Propionibacterium acidipropionici*) are commonly utilized in dairy industry as starters for Swiss cheese ripening (Thierry and Maillard, 2002; Thierry et al., 2005). During this process they ferment lactate and produce propionic and acetic acids and variable amounts of CO_2_ (Thierry and Maillard, 2002; Thierry et al., 2005). Moreover, they are capable to produce vitamins of group B (B9 and B12), conjugated linoleic acid, trehalose, bacteriocins and organic acids, and are known for their probiotic properties and health promoting effects (Cousin et al., 2011; Rabah et al., 2017; Piwowarek et al., 2018). In particular, *P. freudenreichii* modulates the composition of gut microbiota (Bouglé et al., 1999; Seki et al., 2004) due to the production of bifidogenic factors that avoid the settlement of pathobiont *Bacteroides* while favoring the colonization of bifidobacteria (Isawa et al., 2002). Selected strains of *P. freudenreichii* are metabolically active in rat and human gut and show pro-apoptotic properties, mainly due to the production of short chain fatty acids and other molecules such as 1,4-dihydroxy-2-naphthoic acid, that may be beneficial for the treatment and/or prevention of colon cancer, to alleviate the symptoms of colitis, obesity and metabolic syndrome, and to decrease chemotherapy side effects (Lan et al., 2007; Cousin et al., 2011; Deutsch et al., 2017; Do Carmo et al., 2017; Ma et al., 2020; An et al., 2021). *P. freudenreichii* extends the mean lifespan of *C. elegans* by activating the innate immune system via the p38 MAPK pathway, involved in stress response, and the TGF-β pathway associated with anti-inflammation processes in the immune system (Kwon et al., 2016). Moreover, short chain fatty acids and cell wall components, such as surface and cytoplasmatic proteins contribute to *P. freudenreichii* immunomodulatory effect (Rabah et al., 2017). In particular, SlpB and SlpE (surface proteins), and HsdM3, predicted as cytoplasmic protein, are involved in immunomodulation and adhesion to human intestinal epithelial cells (Deutsch et al., 2017; Do Carmo et al., 2017).

All these strain-dependent properties are accompanied by slow growth, efficient utilization of low nutrients concentrations, and the production of an arsenal of proteins involved in multistress perception, adaptation, detoxification, and macromolecules repair (Falentin et al., 2010). Accordingly, *P. freudenreichii* tolerates unfavorable environmental conditions that may be encountered during industrial production or throughout the different gastrointestinal tracts (GITs) of animals and human (Leverrier et al., 2005). Moreover, it exerts antimicrobial activity due to the production of bacteriocins and organic acids (Tharmaraj and Shah, 2009) and antifungal peptides (Lind et al., 2007). All these are useful properties for probiotic and industrial application of this species. Similar to *P. freudenreichii*, also some strains of the species *A. acidipropionici* are considered probiotic for human and animals (Argañaraz-Martínez et al., 2016; Rabah et al., 2017). Based on this evidence, both *P. freudenreichii* and *A. acidipropionici* have been granted the European QPS (Quality Presumption of Safety) and *P. freudenreichii* the American GRAS (Generally Recognized as Safe) status (Cousin et al., 2011; Rabah et al., 2017).

Besides their symbiotic or pro-bioactive properties, another important requirement for novel fermented foods is their economic and environmental sustainability and their production through the valorization of low-cost by-products of the agri-food industry goes in this direction. Scotta also called “secondary cheese whey” or “ricotta cheese exhaust whey,” is one of such by-products. It is obtained by thermal flocculation of whey proteins and contains an average of 5% lactose and about 0.7–1% proteins. Due to its high biological and chemical oxygen demand (BOD and COD about 50,000 and 80,000 mg/L, respectively), it is an environmental high-strength wastewater pollutant whose disposal constitutes a considerable cost for cheese production plants (Sansonetti et al., 2009, 2010; Carvalho et al., 2013; Mostafa, 2013). In addition, other problems encountered in scotta disposal include uneconomical transport due to its high-water content and the difficulty of prolonged storage because of its susceptibility to microbial spoilage. The reverse side of the coin is that scotta retains a nutritional value and is therefore a low-cost substrate for bio-based productions. Accordingly, so far, scotta has been proposed for the extraction of lactose by crystallization (Pisponen et al., 2013), for the production of lactic acid (Secchi et al., 2012), bioethanol (Sansonetti et al., 2009; Zoppellari and Bardi, 2013), sports fermented beverages and synbiotic drinks (Maragkoudakis et al., 2016; Tirloni et al., 2020), bioactive peptides (Monari et al., 2019; Cabizza et al., 2021) and starters for pecorino romano PDO cheese (Chessa et al., 2020). However, still much can be done regarding the exploitation of scotta. Considering that scotta production in Italy reached 750,000 tons in 2019 (Cabizza et al., 2021) and that Sardinia, with an estimate of more than 250,000 tons of scotta per year, is one of the major contributors to the national production of this by-product, the implementation of further processes for its valorization could have important repercussions on the sustainability of the dairy sector.

In this context, the general objectives of this work were the characterization of a library of *Propionibacterium* and *Acidopropionibacterium* (PABs) isolates from goat cheese and milk, and ewe’s rumen and the evaluation of their functional and biotechnological properties for the production of pro-bioactive scotta.

## 2. Materials and methods

### 2.1. Culture media

Media used were the following: modified API50CH medium: Lactose 2%; Tryptone 1%; Yeast Extract 0,5%; K_2_HPO_4_ 250 ppm; MnSO_4_ 50 ppm, Bromocresol purple 170 ppm; YEL: Sodium lactate 1,25%; Tryptone 1%; Yeast Extract 1%; K_2_HPO_4_ 328 ppm; MnSO_4_ 56 ppm (agar 2% when needed); YEL_w/oYE_: as YEL without Yeast Extract; YELactose: Lactose 2%; Tryptone 1%; Yeast Extract 1%; K_2_HPO_4_ 328 ppm; MnSO_4_ 56 ppm (agar 2% when needed); MRS broth (deMan Rogosa and Sharpe medium, VWR, Italy), Iso-Sensitest Agar (Oxoid, Basingstoke, UK). Scotta composition was as follows: Scotta 1: Lactose 4.5%; ash 0.52%; fat 12.5%; protein 0.89%; dry weight 6.38%; pH 6.06. Scotta 2: Lactose 4.0%; ash 2.26%; fat 0.14%; protein 2.15%; dry weight 8.08%; pH 6.23.

### 2.2. Propionibacteria isolation

Isolation of bacteria was carried out from goat cheese (8 samples) and goat milk (12 samples) collected from 3 farms located in east cost of Sardinia and from rumen liquor (9 samples) picked up from ewes in a farm located at Porto Torres as reported by Correddu et al. (2019) and Table 1. For PAB isolation 10 mL g^−1^ of sample was homogenized in a stomacher bag for 5 min and serially diluted in peptone water solution. 100 μL aliquots of the homogenized sample and of each dilution were spread, in double, over YEL medium and incubated for 5 days at 30°C under anaerobic condition (Merck, Microbiology Anaerocult^®^). At end of incubation, propionibacteria typical colonies (beige, red-brownish, yellow, beige-brownish, brown-yellowish, red colonies) were picked and purified in the same medium for further analysis.

**Table 1:** Species and origin of the isolates utilized.

	Origin		
Isolate	Goat cheese	Goat milk	Ewe rumen
*P. freudenreichii*	STAC 3 STAC 4 STAC 4.1 STAC 14 STAC 14.1 STAC 16 STAC 16.1 STAC 32 STAC 42.1 N37 N112	STAC 8/11 STAC 11 STAC 13 STAC 19 STAC 19.1 STAC 35 STAC 38 STAC 40 STAC 42 STAC 46 STAC 47 N17.2 PF2	STAC 5 STAC 7 STAC 10 STAC 25 STAC 30
*A. jensenii*	STAC 1 N24	STAC 45	STAC 23
*A. acidipropionici*	N26 N114 N117	STAC 17.1 STAC 31 STAC 36 N84 N100	STAC 43 N71 N76 N82 SNY
*A. thoenii*		N60	

### 2.3. Isolates identification

Pure cultures were grown under static conditions at 30°C in 10 mL liquid YEL. After 72 h cells were harvested by centrifugation (14,000 rpm for 3 min) and total genomic DNA was extracted with GeneMATRIX Bacterial & Yeast Genomic DNA Purification Kit (EurX, Gdansk, PL) according to the manufacturer instructions, visualized on 1% agarose gel in TBE and quantified spectrophotometrically. Universal primers W001 (5′-AGAGTTTGATCMTGGCTC-3′) and W002 (5′-GNTACCTTGTTACGACTT-3′) were utilized for the amplification of 16S rDNA. PCR reactions were performed on a MyCycler Thermal Cycler System (BioRad. Milano, IT) in 50 μL reaction mixture containing 1 μL template DNA (approximately 20 ng), 1.25 U Taq Polymerase (Promega), 1 × reaction buffer (Mg 2+ free), 2.5 mM MgCl_2_, 200 μM DNTPs, 1.2 μM each of W001 and W002 primers. PCR reactions were run for 35 cycles as follows: denaturation at 94°C for 40 s, annealing at 50°C for 30 s, and elongation at 72°C for 90 s. An initial denaturation step at 94°C for 4 min and a final 7-min extension at 72°C were performed. The PCR products were visualized on a 1% agarose gel in 1 × Tris-borate-EDTA buffer (Chemidoc XRS BioRad, Carlsbad, CA, USA) and quantified spectrophotometrically (Spectrostar nano BMG-Labtech). ExoCleanUp FAST PCR (VWR, Milano, IT) was utilized for the purification of the amplicons prior to DNA sequencing at service facility (Macrogen, Milano, IT). DNA sequencies were aligned by means of BioEdit “sequence alignment editor” (Informer Technologies, Inc.). Sequencies were then compared with those available in GenBank database through BLASTn program.^1^

### 2.4. Physiological characterization

Pure cultures were grown under static conditions at 30°C in 10 mL liquid YEL. After 72 h cells were harvested by centrifugation (14,000 rpm for 3 min), resuspended in sterile water to final OD_600_ = 0.2. For qualitative evaluation of lactose utilization, 24 well microplates containing 990 μL of modified API50CH medium were inoculated with 10 μL of cell suspension prepared as above described. Microplates were incubated under anaerobic conditions (Anaerocult A system, Merck, Darmstadt, Germany) for 7 days at 30°C. The production of acid from lactose was determined based on bromocresol purple color change (de Freitas et al., 2015). For the evaluation of the kinetics of growth on lactose, cells were inoculated to a final OD_650_ = 0.01 in 200 μL YELactose within 96 well microplates. For the assessment of growth kinetics on lactose plus lactate YELactose was added with increasing concentration of Na lactate (0.0, 1.5, 3.0, and 6.0 g L^−1^). Growth was measured automatically every hour at OD_650_ using a SPECTROstar nano microplate spectrophotometer (BMG Labtech, Ortenberg, Germany). Carrying capacity (final OD_650_), specific growth rate (μ), lag phase duration (λ), and area under the curve (AUC) were determined by fitting the growth curves with the Bayesian non-parametric model developed by Tonner et al. (2020).

### 2.5. Characterization for functional properties

Acid tolerance response was evaluated as described by Jan et al. (2000). Briefly, cells grown for 24 h in 10 mL YEL were diluted 1/1,000 into 50 mL YEL. During exponential growth this culture was again diluted 1/1,000 in 50 mL fresh YEL medium. When the culture reached 5 x 10^5^ cells mL^−1^ (OD_650_ = 0.5), bacterial cells were harvested by centrifugation (4,000 rpm x 8 min), resuspended in 50 mL YEL_w/oYE_ pH 4.5 for 30 min at 30°C and centrifuged as before, prior to inoculation, into an equal volume YEL_w/oYE_ pH 2. Residual cell viability was evaluated at time 0 and after 60 min by viable plate count in YEL agar (Jan et al., 2000). For a positive control of growth, the same experiment was carried out in YEL_w/oYE_ medium. Bile salts tolerance was assessed in terms of minimum inhibitory concentration (MIC) of a mixture of 50% Na cholate and 50% Na deoxycholate. Briefly, YEL medium was added with increasing concentration of Bile Salts (SIGMA Aldrich, B8756) ranging from 0.012 to 3.125 mM within 24 well microplates containing 1 mL and inoculated with 10^5^ cells mL^−1^. Microplates were incubated under anaerobic conditions for 48 h after which MIC was determined as the minimum concentration inhibiting growth. Osmotic stress and biofilm formation were assessed as indicated by Deptula et al. (2017) 10 μL cells suspension was inoculated in 190 μL YEL medium containing 1,5%; 3 and 6.5% NaCl (0.3, 0.5, and 1.1 M respectively) within 96 well microplates to a final OD_600_ = 0.05. Growth was measured as OD_595_ after 7 days using a SPECTROstar nano microplate spectrophotometer (BMG Labtech, Ortenberg, Germany). At day 7 all cultures were tested for biofilm formation. To do that planktonic cells were removed by rising microplates twice in tap water. Wells were filled with 100 mL of cristalviolet 1%, incubated for 10 min RT and rinsed repeatedly in cool tap water until water remained clear. Plates were allowed to dry at RT and after dissolving the biofilm with acetic acid 30% under shaking (30 min 300 rpm) cell density was read at OD_540_.

### 2.6. Resistance to lyophilization

Two hundred mL of pure cultures grown in YEL for 72 h at 30°C were harvested by centrifugation (8 min at 4,000 rpm). The resulting cell pellet was taken to −20°C for 24 h and subsequently lyophilized at −50°C and < 0.1 mbar for 48 h with a FreeZone 8 Liter -50C Benchtop Freeze Dryers (Labconco Corporation Kansas City, MO, USA). Viability was evaluated by viable plate count prior to and after lyophilization. For that lyophilized biomass was rehydrated in 200 mL peptone water, after which serial dilutions were plated on YEL Agar plates and viable plate count was carried out after 5 days incubation at 30°C.

### 2.7. Growth kinetics and biomass production in scotta

Pure cultures were grown under static conditions at 30°C in 10 mL liquid YEL. After 72 h cells were harvested by centrifugation (4,000 rpm for 8 min), resuspended in 10 mL sterile water, inoculated in 120 mL scotta to a final concentration of 1 × 10^6^ cell mL^−1^ and incubated for 72 h at 30°C under static conditions. Viable plate count was carried out on YEL Agar plates at time 0 (inoculum) and after 24, 48, and 72 h. Two technical replicates of two biological replicates were carried out on two different scotta sampled in two dairy industries located in north-west Sardinia.

### 2.8. Analytical methods

Mean chemical composition of scotta was analyzed according to the method described by Cabizza et al. (2021). Acetic and propionic acids analysis was carried out by solid phase micro extraction followed by gas chromatography mass spectrometry. Briefly, 1.0 mL of scotta sample were placed into a 20 mL SPME vial (75.5 × 22.5 mm) that was tightly closed using a septum. After 10 min of equilibration at 40°C, a 100 μm PDMS/DVB/CAR (Polydimethylsiloxane/Divinylbenzene/Carboxen) coated fiber (Supelco, Sigma Aldrich, St. Louis, MO, USA) was injected through the septum and suspended in the headspace. The fiber was exposed to the volatiles for 20 min; it was then retracted, removed from the vial, and placed immediately into the injector of the GC. Thermal desorption was performed in the injector at a temperature of 250°C for 5 min in splitless injection mode. Prior to and after each analysis, the fiber underwent a further bake-out step for 5 min at 250°C. The chromatographic separation was accomplished using the following temperature program: 40°C hold for 4 min, then increased to 150°C at a rate of 5.0°C min^−1^, held for 3 min, then increased to 240°C at a rate of 10°C min^−1^, and finally held for 12 min. Helium was used as the carrier gas at a constant flow of 1 mL min^−1^. Quantification of acetic acid and propionic acids was accomplished by means an external calibration on three levels of concentration. A stock solution of each standard was prepared by weighting it accurately into a 10 mL volumetric flask. The resulting stock solution was diluted with scotta free of acetic and propionic acid in order to obtain three reference solutions at decreasing concentrations. For each compound the calibration curves were made by linear regression by plotting the peak area of external standard against their known concentrations. Both compounds were quantified by a selected ion monitoring (SIM) method setting the quadrupole to filter the ions 60 for acetic acid and 73 and 74 for propionic acid. Each analysis was performed in duplicate, and the results were expressed as g L^−1^.

For the untargeted metabolomics analysis, fermented and non-fermented scotta samples were thawed at RT, and then 1 mL aliquots were extracted by using 4 mL of a methanol/water (both LC -MS grade, from Sigma-Aldrich) 50/50 solution (vol/vol) according to an ultrasound-assisted extraction (10 min at maximum power, 120 Watt) to disrupt microbial cells. After that, samples were vortexed and centrifuged at 4,500 g for 10 min to remove large biomolecules, such as proteins. Supernatants were filtered through 0.22 μm cellulose syringe filters in UHPLC vials for further HRMS targeted analysis. The untargeted UHPLC-HRMS analysis was done using a Q-Exactive Focus Hybrid Quadrupole-Orbitrap Mass Spectrometer (Thermo Scientific) coupled to a Vanquish UHPLC pump and equipped with heated electrospray ionization-II probe (Thermo Scientific; Rocchetti and O’Callaghan, 2021). The chromatographic separation was based on a water–methanol (both liquid chromatography-MS grade, from Sigma-Aldrich) gradient elution (0.5–98% acetonitrile in 12 min), using 0.1% formic acid as phase modifier, and using an Agilent Zorbax Eclipse Plus C18 column (50 × 2.1 mm, 1.8 μm). The column was re-equilibrated up to 18 min with 99.5% water. The flow rate was 200 μL min^−1^, and the injection volume was 6 μL. The mass spectrometer was calibrated using Pierce TM positive ion calibration solution (Thermo Fisher Scientific). The H-ESI parameters were the following: sheath gas 40 (arb), auxiliary gas (20), spray voltage 3.5 kV, capillary temperature 320°C, S-lens RF level 50, auxiliary gas heater temperature 50°C. The HRMS was done in the Full MS1 isotopic method (resolution 70,000 FWHM), in the range 300–1,600 m/z, setting the following scan parameters: AGC target 1e^6^ and maximum injection time (IT) 50 ms. The raw data generated by UHPLC-HRMS were then processed using the software MS-DIAL (version 4.70). The putative annotation (level 2 of confidence typical of untargeted metabolomics-based experiment) was reached via spectral matching against the comprehensive databases FooDB^2^, using a tolerance for mass accuracy of 5 ppm. For the semi-quantification of cobalamin derivatives and vitamers of folic acid, we used standard solutions of vitamin B12 (cyanocobalamin) and folic acid (B9) (both purchased from Sigma-Aldrich) at different concentration levels (namely: 500, 250, 50, 25, and 2.5 μg L^−1^), considering coefficients of determination (R^2^) > 0.98.

### 2.9. Antibiotic susceptibility

Minimum inhibitory concentrations (MIC) of different antibiotics were determined using broth microdilution method according to the protocol ISO 10932:2010. In particular, bacterial isolates were inoculated in 96 microwell plates containing serial two-fold dilutions of the following antibiotics: ampicillin, amoxicillin, ciprofloxacin, clindamycin, chloramphenicol, erythromycin, gentamicin, streptomycin, kanamycin, spectinomycin, tetracycline, trimethoprim/sulfamethoxazole and vancomycin. The MIC values obtained were interpreted according to the criteria proposed by EFSA (2012). For trimethoprim/sulfamethoxazole the CLSI break point proposed from *Corynebacterium* spp. and *Lactococcus* spp. ≥ 4/76 was considered. For spectinomycin the ECOFF of *Staphylococcus aureus* (≥ 128) was considered. For ciprofloxacin the CLSI break point for Gram positive anaerobes (CLSI, 2018) were used MIC90 and MIC50 were evaluated as the lowest concentration of the antibiotic at which 90 and 50% of the isolates were inhibited, respectively.

### 2.10. Statistical analyses

All experiments were carried out in triplicate (at least four technical replicates of tree biological replicates) unless otherwise stated. Data were subject to analysis of variance (Anova) and Tuckey HSD post-hoc test. Additionally, the software MetaboAnalyst 5.0^3^ and SIMCA 17 (Umetrics, Sweden) were used for multivariate statistics (unsupervised hierarchical clustering and supervised OPLS-DA) to extrapolate the biomarker compounds (VIP) and their Fold-Change (FC) variations against the non-fermented samples.

## 3. Results

### 3.1. Isolation and identification of propionibacteria

Forty-seven isolates showing PAB typical colony (beige, red-brownish, yellow, beige-brownish, brown-yellowish, red colonies) on YEL agar plates were obtained from 29 samples of goat milk and cheese and from ewe’s rumen liquor sampled in three different areas of Sardinia. Molecular identification of the isolates, based on BLAST analysis of 16S rDNA sequencies, led to their identification as *Propionibacterium* and *Acidopropionibacterium* spp. Twenty-nine isolates (corresponding to 62%) were ascribed to *P. freudenreichii*, most of them coming from milk (45%) and cheese (38%) with 5 isolates (17%) from rumen liquor. Of the remaining isolates, 13 (26.5%) were identified as *A. acidopropionici*, 4 as *A. jensenii* and 1 as *A. thoenii* (Table 1).

### 3.2. Assessment of antibiotic susceptibility

The MICs of 13 antibiotics, nine of which recommended by EFSA, were evaluated according to protocol ISO 10932:2010. The distribution of the MICs differed between *P. freudenreichii* and *Acidipropionibacterium* spp. for all tested antimicrobials, except for amoxicillin to which all isolates proved resistant (Tables 2, 3). *P. freudenreichii* isolates appeared susceptible to ampicillin, vancomycin (with just one isolate showing a MIC of 8 mg/L), clindamycin, gentamycin, and streptomycin (EFSA, 2008). Eighty-two percent of the isolates could be considered susceptible to chloramphenicol. Ciprofloxacin exerted limited effect on PABs since 48% of the isolates were resistant with a MIC>2 mg/L (CLSI break point for Gram positive anaerobes). Four isolates proved resistant to kanamycin with a MIC>64 mg/L (EFSA, 2008) and 13% to tetracycline (EFSA ECOFF >2 mg/L). For erythromycin, 24% of the isolates appeared resistant according to the current EFSA ECOFF. Most of the isolates (83%) could be considered susceptible trimethoprim/sulfamethoxazole, according to CLSI break point for *Corynebacterium* spp. and *Lactococcus* spp., while 71% of them were resistant to spectinomycin (Table 2 and Supplementary Table 2). Regarding *Acidipropionibacterium* spp., all of them proved susceptible to ampicillin, chloramphenicol and clindamycin (except for one isolate), gentamycin, streptomycin, trimethoprim/sulfamethoxazole, and vancomycin. Seven of them appeared resistant to tetracycline, three to ciprofloxacin and 2 to kanamycin (Table 3). Overall, MIC90 of *P. freudenreichii* isolates was higher than that of *Acidipropionibacterium* spp. for 6 antibiotics tested (CHL, ERY, KAN, SPE, STR and VAN) and lower for three antibiotics (Supplementary Table 1). Moreover, four *P. freudenreichii* (STAC 13, STAC 16, STAC 47, N17.2) had three antibiotic resistances and two *Acidipropionibacterium* spp. (STAC 17.1 and N26) presented four antibiotic resistances. On the contrary, no antibiotic resistances were reported for three *P. freudenreichii* (STAC 7, STAC 19, N112) and six *Acidipropionibacterium* spp. (STAC 36, N24, N84, N117 and SNY) isolates.

**Table 2:** *Propionibacterium freudenreichii* antibiotic susceptibility.

Isolate	AMP (mg L^−1^)	AMX (mg L^−1^)	CHL (mg L^−1^)	CIP (mg L^−1^)	CC (mg L^−1^)	ERY (mg L^−1^)	GENT (mg L^−1^)	KAN (mg L^−1^)	SPE (mg L^−1^)	STR (mg L^−1^)	TET (mg L^−1^)	T/S (mg L^−1^)	VAN (mg L^−1^)
STAC 3	0.5	>128	2	4	0.0625	0.03125	4	64	>256	8	1	2/38	1
STAC 4	2	>128	2	2	0.0625	0.25	2	256	>256	32	0.5	0.0625/1.1875	1
STAC 4.1	0.0625	>128	2	4	0.125	0.0625	0.5	32	>256	2	0.5	0.0625/1.1875	8
STAC 5	0.5	>128	4	2	0.125	0.5	2	16	64	1	0.5	2/38	2
STAC 7	0.5	>128	2	2	0.25	0.03125	8	16	>256	16	0.5	0.25/4.75	2
STAC 8/11	0.5	>128	2	4	0.0625	0.0625	0.5	16	>256	1	0.5	0.0625/1.1875	1
STAC 10	0.5	>128	2	2	0.0625	4	8	64	64	16	0.5	0.25/4.75	0.5
STAC 11	0.125	>128	1	2	0.0625	0.0625	1	32	>256	1	0.25	0.03125/0.593	1
STAC 13	1	>128	1	64	0.125	8	2	64	8	2	4	0.0625/1.1875	0.5
STAC 14	0.5	>128	2	4	0.5	0.03125	0.5	16	>256	4	0.25	0.0625/1.1875	1
STAC 14.1	0.0625	>128	1	4	0.0625	0.03125	4	32	>256	2	0.5	2/38	1
STAC 16	1	>128	2	8	0.0625	0.5	32	4	>256	32	32	8/152	1
STAC 16.1	0.5	>128	2	2	4	0.5	32	8	>256	32	1	8/152	1
STAC 19	0.03125	>128	1	4	0.03125	0.03125	2	16	>256	4	0.25	0.125/2.375	1
STAC 19.1	0.5	>128	2	2	0.03125	0.0625	1	8	16	2	4	0.25/4.75	1
STAC 25	0.25	>128	4	2	0.03125	0.03125	8	64	>256	16	0.5	0.5/9.5	0.5
STAC 30	0.03125	>128	2	4	0.125	0.0625	0.5	32	>256	2	0.5	0.03125/0.593	0.5
STAC 32	1	>128	2	4	0.0625	0.25	1	256	>256	32	0.5	0.0625/1.1875	1
STAC 35	0.5	>128	2	4	0.03125	0.125	0.5	8	>256	4	1	0.0625/1.1875	1
STAC 38	0.5	>128	2	0.5	0.03125	0.5	0.5	4	8	1	0.25	0.25/4.75	0.5
STAC 40	0.5	>128	4	2	0.125	2	4	32	128	4	1	4/76	2
STAC 42	0.25	>128	2	4	0.125	0.5	8	256	>256	32	0.5	0.015625/0.295	1
STAC 42.1	2	>128	2	2	0.0625	1	8	64	16	8	2	0.5/9.5	0.5
STAC 46	0.03125	>128	0.5	4	0.0625	1	4	64	>256	4	0.5	0.125/2.375	1
STAC 47	0.125	>128	4	0.25	0.125	>8	0.5	2	64	1	64	0.25/4.75	1
N17.2	0.5	>128	1	8	0.03125	0.5	1	256	>256	0.5	1	0.0625/1.1875	1
N37	0.125	>128	2	4	0.125	0.0625	0.5	64	>256	2	0.5	0.0625/1.1875	1
N112	0.5	>128	1	2	0.0625	0.0625	8	32	>256	8	1	0.125/2.375	0.5
PF2	0.5	>128	4	2	0.0625	32	2	16	>256	2	1	0.0625/1.1875	1

**Table 3:** *Acidipropionibacterium* spp. antibiotic susceptibility.

Isolate	AMP (mg L^−1^)	AMX (mg L^−1^)	CHL (mg L^−1^)	CIP (mg L^−1^)	CC (mg L^−1^)	ERY (mg L^−1^)	GENT (mg L^−1^)	KAN (mg L^−1^)	SPE (mg L^−1^)	STR (mg L^−1^)	TET (mg L^−1^)	T/S (mg L^−1^)	VAN (mg L^−1^)
STAC 1	0.03125	>128	0.5	2	0.03125	0.0625	0.5	32	8	1	1	0.0625/1.1875	0.5
STAC 17.1	0.25	>128	2	4	0.25	8	32	256	>256	32	1	1/19	1
STAC 23	0.5	>128	2	4	0.0625	0.0625	8	8	8	2	4	0.0625/1.1875	
STAC 31	1	>128	1	1	nd	0.0625	2	8	16	2	4	0.0625/1.1875	0.5
STAC 36	1	>128	1	1	0.0625	0.125	2	16	32	2	2	0.0625/1.1875	1
STAC 43	1	>128	0.5	0.25	0.0625	0.0625	1	8	8	1	4	0.0625/1.1875	0.25
STAC 45	0.5	>128	0.5	0.5	0.0625	0.25	2	8	8	2	8	0.0625/1.1875	0.5
N24	0.5	>128	2	1	0.25	0.03125	1	16	16	2	2	0.125/2.375	0.5
N26	2	>128	4	2	0.0625	2	8	128	64	16	4	0.5/9.5	1
N60	0.5	>128	2	0.5	0.5	0.0625	0.5	8	1	1	2	0.0625/1.1875	0.25
N71	1	>128	2	1	0.0625	0.0625	1	4	8	1	4	0.0625/1.1875	0.5
N76	1	>128	1	0.5	0.0625	0.03125	1	16	8	0.5	0.5	0.0625/1.1875	0.5
N82	2	>128	0.5	1	0.0625	0.0625	1	8	8	1	4	0.0625/1.1875	0.25
N84	0.5	>128	2	1	0.0625	0.0625	1	8	16	1	2	0.0625/1.1875	0.25
N100	1	>128	0.5	1	0.125	0.0135	1	8	16	16	2	0.03125/0.593	0.5
N114	1	>128	2	8	0.125	2	8	16	>256	16	1	4/76	1
N117	1	>128	2	1	0.0625	0.0625	4	32	8	4	1	2/38	0.5
SNY	0.5	>128	1	0.5	0.125	0.0625	1	8	4	1	1	0.0625/1.1875	0.5

### 3.3. Growth on lactose containing medium

**Figure 1:**
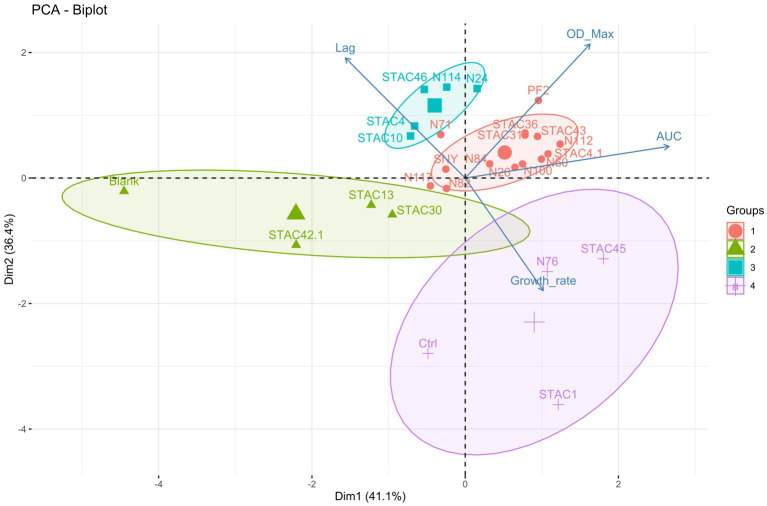
Principal component analysis (PCA) biplot of strains and growth variables. The magnitude of the vectors shows the strength of their contribution to each PC. Vectors pointing in similar directions indicate positively correlated variables, vectors pointing in opposite directions indicate negatively correlated variables, and vectors at proximately right angles indicate low or no correlation. Colored concentration ellipses (size determined by a 0.95-probability level) show the observations grouped by mark class as determined by K-means clustering algorithm.

Since lactose is the main carbon source in scotta, the 47 PABs were inoculated on modified API 50CH medium to evaluate lactose fermentation ability. At first, the acidification, and consequent color change of the medium from purple to red, orange and yellow, corresponding to no acidification (−), low (+), average (++) and high acidification (+++), respectively, was evaluated. Twenty-two isolates (21 *P. freudenreichii* and one *A. acidipropionici*), that showed no medium acidification, were considered uncapable of lactose fermentation, and therefore excluded from the present screening. Of the remaining 25 isolates that showed various extent of medium acidification, 9 were ascribed to the species *P. freudenreichii*, 12 to *A. acidopropionici*, 3 to *A. jensenii* and 1 to *A. thoenii*. All these isolates were assessed for their growth kinetics on YELactose. To do that 200 growth curves were obtained and fitted by the growth model of Tonner et al. (2020) to evaluate carrying capacity, lag phase duration (λ), growth rate (μ) and area under the curve (AUC). This last parameter integrates the contributions of the lag phase duration, growth rate, and carrying capacity into a single value, thus somewhat summarizing the growth curve. As reported in Table 4, *A. jensenii* STAC 1 showed the highest growth rate (*μ* = 0.616 ± 0.044 h^−1^) and the shortest lag phase (*λ* = 9.0 ± 1.0 h). Isolates *A. jensenii* STAC 45, *A. acidipropionici* N76 and *P. freudenreichii* STAC 10, and STAC 4 showed a relatively high growth rate, ranging from 0.19 to 0.29 h^−1^, and differed greatly in lag phase duration (from 4.1 ± 4.8 h in STAC 45 to 73.1 ± 3.5 h in STAC 4). The remaining isolates showed poor growth on this medium with μ ranging from 0.018 h^−1^ to 0.12 h^−1^ and variable lag phase duration. Accordingly, generation time (g), that under optimal conditions is about 5–6 h, varied markedly among strains ranging from 1.109 ± 0.02 to 30.37 ± 0.53 h (data not shown). Most of the isolates showed comparable OD_650_, except for the *A. acidipropionici* N114, STAC 36, STAC 43, and *P. freudenreichii* STAC 46, that showed the best performances on YELactose (FDR adjusted *p* value < 0.05). *Acidipropionibacterium* spp. N100, N60, STAC 45 and *P. freudenreichii* N112 displayed the higher AUC values, followed by *Acidipropionibacterium* spp. N117, N24, N26, N76, N82, N84, STAC1, STAC 31, STAC 36, and STAC 43. The lowest AUC values were measured in *Acidipropionibacterium* spp. N71, N117 and in *P. freudenreichii* STAC 46, STAC 4, and STAC 42.1. Based on these parameters, all the isolates were subdivided into four clusters by the k-means algorithm (Figure 1). Most of them (namely *P. freudenreichii* N112, PF2, STAC 4.1 and *Acidipropionibacterium* spp. N24, N26, N60, N84, STAC 31, STAC 36, and STAC 43) were included in cluster 1 and showed a slow average growth rate (0.095 h^−1^) and an intermediate lag phase duration (34.87 h) but reached a high carrying capacity (1.383 OD_650_) and the highest AUC (258.53). On the contrary, the three isolates grouped in cluster 2 (*A. acidipropionici* N76 and *A. jenseni* STAC 1, and STAC 45) adapted rapidly (average λ = 7.46 h) and grew fast (average μ = 0.387 h^−1^) on YELactose but reached a lower AUC (247.47) due to a lower carrying capacity (0.847 OD_650_). Isolates in cluster 3 (*A. acidpropionici* N114, N71 and *P. freudenreichii* STAC 10, STAC 4, and STAC 46) were characterized by a higher carrying capacity (OD_650_ = 1.402) with a low AUC (148.105) because of the most extended lag phase (average *λ* = 65.74 h). Lastly, strains in cluster 4 (*A. acidpropionici* N117, N82, SNY, and *P. freudenreichii* STAC13, STAC30, and STAC 42.1) performed poorer in YELactose with an overall AUC of 143.55 that resulted from the slowest growth rate (average *μ* = 0.056 h^−1^), carrying capacity (OD_650_ = 0.7954) and long lag phase (average *λ* = 30.56 h).

**Table 4:** Growth kinetics in YELactose.

Strain	Carrying capacity (OD_650_)	Growth rate (h^−1^)	Lag phase (h)	Area Under the Curve (AUC)
STAC 1	0.35 ± 0.06 h	0.616 ± 0.044 a	9.0 ± 1.00 i	244 ± 90 def
STAC 4	1.46 ± 0.09 ab	0.302 ± 0.022 b	73.1 ± 3.50 a	138 ± 12 k
STAC 4.1	1.46 ± 0.03 ab	0.115 ± 0.006 d	24.8 ± 1.20 h	254 ± 10 cde
STAC 10	1.47 ± 0.03 ab	0.295 ± 0.038 b	71.2 ± 1.0 a	161 ± 7 ii
STAC 13	0.53 ± 0.03 g	0.026 ± 0.001 fg	36.6 ± 4.0 ef	158 ± 2 ii
STAC 30	0.62 ± 0.05 g	0.031 ± 0.004 efg	27.4 ± 1.4 gh	173 ± 9 hi
STAC 31	1.48 ± 0.04 ab	0.106 ± 0.011 d	34.9 ± 4.8 fg	211 ± 10 fg
STAC 36	1.54 ± 0.01 a	0.120 ± 0.001 d	33.6 ± 0.5 fg	232 ± 3 efg
STAC 42.1	0.28 ± 0.02 h	0.018 ± 0.003 g	52.9 ± 12.8 c	72 ± 6 L
STAC 43	1.51 ± 0.04 ab	0.114 ± 0.009 d	32.0 ± 2.8 fg	229 ± 6 efg
STAC 45	1.14 ± 0.06 f	0.239 ± 0.063 bc	4.1 ± 4.8 i	281 ± 11 abc
STAC 46	1.53 ± 0.05 ab	0.120 ± 0.012 d	65.0 ± 2.1 ab	163 ± 42 ii
N24	1.43 ± 0.02 ab	0.096 ± 0.002 d	24.7 ± 0.6 h	246 ± 9 def
N26	1.34 ± 0.22 cd	0.079 ± 0.016 def	30.0 ± 1.4 gh	270 ± 8 bcd
N60	1.2 ± 0.04 ef	0.081 ± 0.002 de	27.9 ± 1.1 gh	289 ± 2 ab
N71	1.4 ± 0.04 abc	0.091 ± 0.007 d	41.8 ± 2.0 de	154 ± 14 jk
N76	1.34 ± 0.03 cde	0.197 ± 0.090 c	9.4 ± 6.5 i	217 ± 23 efg
N82	1.36 ± 0.05 bcd	0.086 ± 0.026 de	26.1 ± 3.6 gh	224 ± 5 efg
N84	1.34 ± 0.07 cd	0.104 ± 0.009 d	27.6 ± 2.9 gh	195 ± 23 gh
N100	1.43 ± 0.06 abc	0.083 ± 0.026 de	27.4 ± 1.4 gh	311 ± 17 a
N112	1.35 ± 0.07 cd	0.073 ± 0.002 def	26.6 ± 1.9 gh	298 ± 16 ab
N114	1.54 ± 0.05 a	0.076 ± 0.007 def	57.6 ± 2.7 bc	156 ± 3 ii
N117	1.31 ± 0.05 de	0.085 ± 0.029 de	30.5 ± 4.1 gh	222 ± 34 efg
PF2	1.52 ± 0.04 ab	0.116 ± 0.017 d	49.6 ± 2.8 cd	290 ± 3 ab
SNY	1.23 ± 0.13 ef	0.092 ± 0.015 d	30.9 ± 3.1 gh	156 ± 20 ii

Results are mean ± std of four technical replicates of two biological replicates. Different letters in the same column indicate values significantly different as determined by Tukey-HSD test (*p* < 0.05).

### 3.4. Functional properties of lactose fermenting PABs

Functional properties of the 25 PABs showing growth on YELactose were assessed *in vitro* in terms of resistance to bile salts, and to acid and osmotic stress. To assess bile salts resistance, the 25 PABs isolates were challenged with increasing concentrations of unconjugated bile salts (from 0.012 to 3.125 mM). More than half of the isolates proved resistant to 1.56 mM and 10 of them resisted 0.78 mM unconjugated bile salts (Table 5). In parallel, acid stress tolerance of the 25 isolates was evaluated. Acidification is a common practice in the food industry to prevent spoilage by alterative or pathogenic microorganisms. Moreover, fermented food bacteria transit through the stomach where they are exposed to pH values ranging from 1 and 2. Since *Propionibacterium* spp. acid stress tolerance increases after the exposure to a sub-lethal acid stress (Jan et al., 2000), all isolates were kept at pH 4.5 for 30 min before being transferred to pH 2.0 for 60 min. *P. freudenreichii* N114, N76 and *A. acidipropionici* N84, did not survive sub-acid pre-treatment. After 60 min at pH 2 *Acidipropionibacterium* spp. STAC 1, STAC 23, STAC 31, STAC 36, N24, N26, N60, N71, N82, N100, N117 showed <1% viability while STAC 43, STAC 45, and *P. freudenreichii* STAC 30, STAC 46, N112 and PF2 showed <50% viability. The remaining four isolates, namely STAC 4, STAC 4.1, STAC 10 and STAC 42.1, showed a remarkable resistance to acid stress (Table 5) with an average of Log 9.00 ± 0.21 CFU mL^−1^ after 60 min at pH 2.0 (Table 6). These four isolates were therefore subjected to further characterization. As for osmostress resistance, NaCl showed a dose dependent effect on overall growth, although with some significant differences among strains. *P. freudenreichii* STAC 4.1 and STAC 42.1 proved the best growers for NaCl concentrations up to 3%. At 6.5% none of them overcome OD_595_ = 0.6 (Table 7). Limited biofilm formation was observed solely in *P. freudenreichii* STAC 4.1 at 6.5% NaCl.

**Table 5:** Bile salts and acid stress resistance.

Strain	Bile salt resistance (mM)	Acid stress resistance (%)
STAC 1	1.56	<1
STAC 4	0.78	>50
STAC 4.1	1.56	>50
STAC 10	1.56	>50
STAC 23	0.78	<1
STAC 30	0.78	<50
STAC 31	1.56	<1
STAC 36	1.56	<1
STAC 42.1	0.78	>50
STAC 43	1.56	<50
STAC 45	0.78	<50
STAC 46	1.56	<50
N24	0.78	<1
N26	1.56	<1
N60	0.78	<1
N71	1.56	<1
N76	0.78	Ns
N82	1.56	<1
N84	1.56	Ns
N100	1.56	<1
N112	0.78	<50
N114	0.78	Ns
N117	1.56	<1
PF2	1.56	<50
SNY	1.56	<1

Bile salts resistance is expressed in terms of MIC of unconjugated bile salts. Acid stress resistance is expressed in terms percentage of residual cell viability after 30 min pre-treatment at pH 4.5 and 60 min treatment at pH 2. Ns indicates isolates not surviving pre-treatment at pH 4.5. Results are representative of at least four technical replicates of three biological replicates.

**Table 6:** Viable plate counts following 60 min at pH 2.0.

Isolate	Log CFU mL^−1^ t0	Log CFU mL^−1^ t1
STAC 4	8.819 ± 0.026 b	8.910 ± 0.235 bc
STAC 4.1	8.265 ± 0.039 c	8.508 ± 0.014 c
STAC 10	9.454 ± 0.037 a	9.113 ± 0.023 ab
STAC 42.1	9.416 ± 0.055 a	9.500 ± 0.460 a

Results are mean ± std of four technical replicates of three biological replicates. Different letters in the same column indicate values significantly different as determined by Tukey-HSD test (*p* < 0.01).

**Table 7:** Osmotic stress resistance and biofilm formation.

	Isolate	NaCl 0%	NaCl 1.5%	NaCl 3%	NaCl 6.5%
Osmotic stress resistance	STAC 4	0.867 ± 0.066 c	0.737 ± 0.027 b	0.690 ± 0.028 c	0.444 ± 0.015 a
	STAC 4.1	1.113 ± 0.116 b	1.318 ± 0.240 a	0.873 ± 0.026 b	0.363 ± 0.022 b
	STAC 10	0.906 ± 0.020 c	0.824 ± 0.211 b	0.712 ± 0.042 c	0.544 ± 0.193 a
	STAC 42.1	1.369 ± 0.062 a	1.173 ± 0.084 a	1.016 ± 0.061 a	0.436 ± 0.020 a
Biofilm formation	STAC 4	0.172 ± 0.054 a	0.210 ± 0.043 a	0.224 ± 0.030 a	0.099 ± 0.091 b
	STAC 4.1	0.216 ± 0.035 b	0.180 ± 0.019 bc	0.111 ± 0.018 c	0.513 ± 0.129 a
	STAC 10	0.299 ± 0.057 a	0.201 ± 0.031 b	0.198 ± 0.057 b	0.183 ± 0.059 b
	STAC 42.1	0.238 ± 0.072 a	0.248 ± 0.044 a	0.285 ± 0.083 a	0.294 ± 0.030 a

Osmotic stress resistance and biofilm formation were evaluated after 7 days on YEL w/o (0%) and with increasing concentrations of NaCl. Osmotic stress resistance is expressed as OD_595_. Biofilm formation is expressed as OD_540_. Results are mean ± std of at least 5 technical replicates of three biological replicates. Different letters in the same column indicate values significantly different (*p* < 0.05).

### 3.5. Biotechnological properties of selected PABs

**Figure 2:**
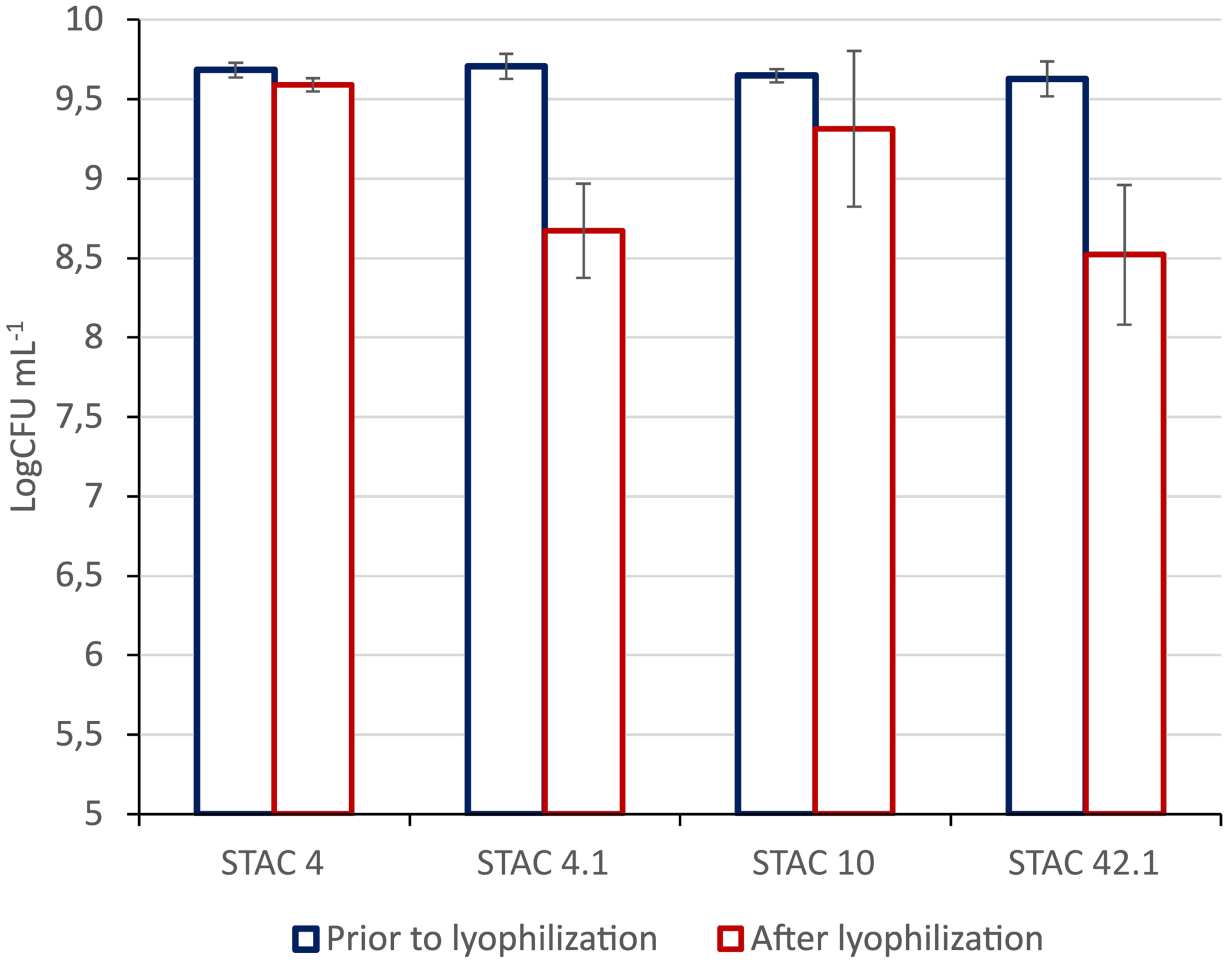
Resistance to lyophilization. Viable Counts are expressed as Log CFU mL^−1^ prior to and after lyophilization. Results are media ± std of at least three technical replicates of three biological replicates.

Lactate is the preferred carbon source for dairy PABs (Piveteau, 1999). To evaluate to which extent lactate improves growth in lactose containing medium, growth kinetics of STAC 4, STAC 4.1, STAC 10 and STAC 42.1 were assessed on YELactose without (0) and with 1.5, 3 and 6 g L^−1^ lactate. The effects of the inoculated strain (first independent variable), and of the concentration of lactose (second independent variable) on maximum cell density, growth rate and duration of lag phase (dependent variables) were evaluated by two-way ANOVA. As expected, PABs cell density significantly (*p* < 0.05) increased with lactate concentration, although in a strain dependent fashion (Figure 2). Particularly, the Tukey’s HSD post-hoc test indicated that the shift from 0 g L^−1^ to 1.5 g L^−1^ of lactate were sufficient to significantly (*p* < 0.05) increase final cell density. This effect was particularly evident for STAC 42.1, that, although reaching the lowest cell density in all condition tested, more than tripled OD_650_ when going from 0 to 1.5 g L^−1^ lactate. Regarding the growth rate, this was not significantly affected (*p* = 0.960) by the addition of lactate to the culture media. On average, lag phase duration significantly (*p* < 0.05) decreased from 50.2 to 30.7 h in the presence of lactate. The strongest reduction (4.3 fold) was observed in STAC 42.1 that showed the lowest lag phase in the presence of lactate.

**Figure 3:**
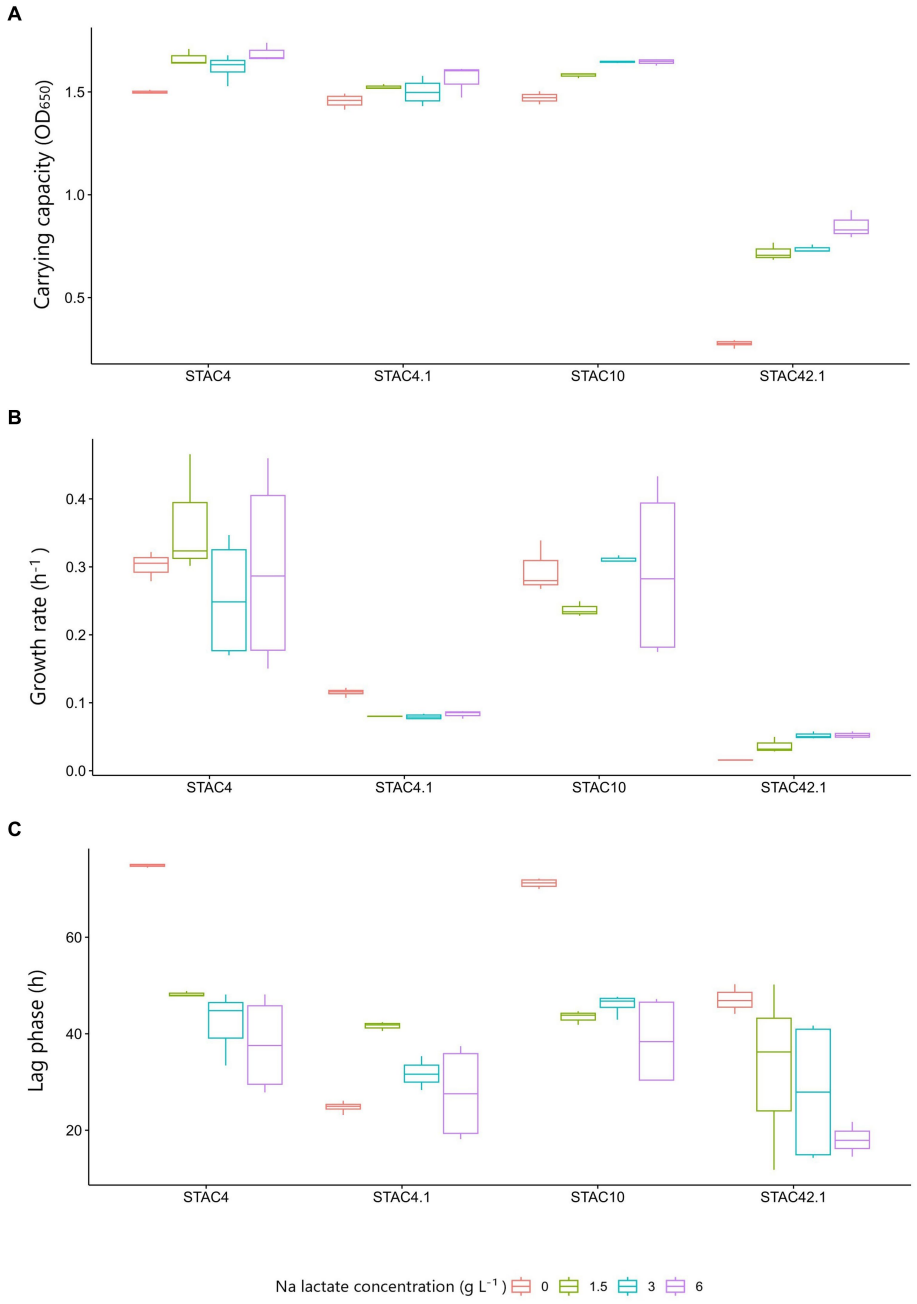
Growth parameters (**A**, Carrying capacity; **B**, Growth rate; **C**, Lag phase) during growth in lactose containing medium w/o (0) and with increasing concentrations (1.5, 3, and 6 g L^−1^) of Na lactate. Results are media ± std of at least three technical replicates of three biological replicates.

Since it is commonly utilized for the stabilization of probiotics in novel foods, the impact of lyophilization on cell viability was also evaluated. As shown in Figure 3, residual viability after lyophilization ranged from 88 to 99%, although no membrane stabilizers were added prior to the treatment.

### 3.6. Growth kinetics and metabolomics profiling in scotta

**Figure 4:**
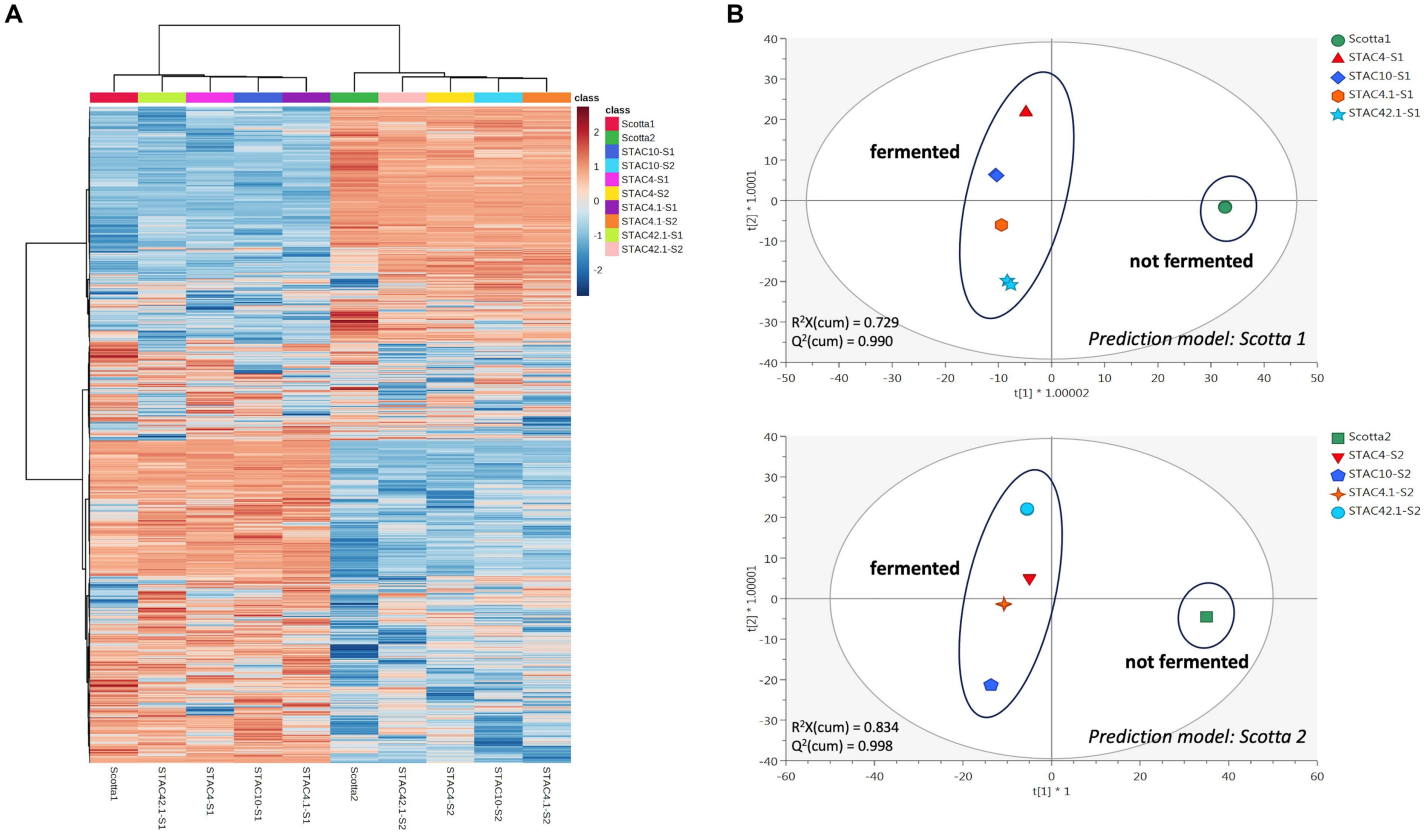
Unsupervised hierarchical clustering heat map **(A)** and Orthogonal Projections to latent Structures discriminant analyses **(B)**, considering the untargeted metabolomic profile of the fermented and not-fermented scotta samples.

STAC 4, STAC 10, STAC 4.1 and STAC 42.1 were inoculated in two scotta samples differing in pH and composition and coming from two dairy industries differing in the technological level. Growth monitoring for 72 h revealed that in scotta the four isolates reached the stationary phase after 48 h, although differing in final cell density (Table 8 and Supplementary Figure 1). In scotta 1, the best and the least growers were STAC 4 and STAC 42.1 that reached 9.45 and 8.89 Log CFU mL^−1^, respectively. In scotta 2, STAC 4 and STAC 10 reached comparable cell densities (9.15 and 9.12 Log CFU mL^−1^, respectively) while STAC 4.1 and STAC 42.1 stopped growth at lower cell densities (about 8.11 and 8.57 Log CFU mL^−1^, respectively). Propionic and acetic acids production were in accordance with growth performances. Thus, STAC 4 and STAC 42.1 were the best and the least propionic acid producers, and the same trend was observed also for acetic acid (Table 8). P/A molar ratio was generally higher than that found on lactose containing medium by Unigunde et al. (2023) and comparable on the two scotta for all isolates except for STAC 42.1. This showed the highest P/A ratio, reaching 4.61 in scotta 1 (Table 8). The metabolomics approach revealed a clear matrix effect, likely due to inherent differences in technological processing between the two scotta samples under investigation. Figure 4A (i.e., heat map) clearly divided the scotta samples in two clusters according to the scotta considered, while highlighting also different performances of the selected isolates during the fermentation. Besides, the supervised prediction models (OPLS-DA score plots) reported in Figure 4B allowed to confirm the clear modifications of the chemical profiles due to the fermentation step, thus providing the hyperspace separation between and within groups. The UHPLC-HRMS profiling approach also allowed the semi-quantification of both vitamin B9 vitamers and cobalamin-derivatives in both scotta samples (Table 8), although a matrix effect could be clearly noticed. The folates content ranged between 0.03 (STAC 42.1 in scotta 2) and 0.16 mg L^−1^ (STAC 10 in scotta 2). Regarding cobalamin-derivatives (vitamin B12), the lowest range was recorded for STAC 4 in scotta 1 (0.49 mg L^−1^), while the isolates performed better in scotta 2 (Table 8), recording an average content of 1.28 mg L^−1^. Finally, the prediction ability of the different vitamers was extrapolated for the prediction models reported in Figure 4B when considering both scotta 1 and scotta 2. It was interesting to notice that for both models, a higher prediction ability was observed for folate derivatives; the most predictive and discriminant metabolite following the fermentation of scotta 1 was 5-Methyl-THF (VIP score = 1.55), while Dihydrofolic acid (DHF) was the most discriminant compound of the fermented scotta 2 (VIP score = 1.33). The VIP scores and the LogFC variations recorded vs. the non-fermented scotta samples are reported in Supplementary Table 3.

**Table 8:** Analyses of fermented scotta.

	Strain	Log CFU mL^−1^	Acetic acid (g L^−1^)	Propionic acid (g L^−1^)	P/A	Total Folate Eq. (mg L^−1^)	Total Cobalamin Eq. (mg L^−1^)
Scotta 1	STAC 4	9.45 ± 0.06 a	1.00 ± 0.037 a	3.34 ± 0.223 a	3.34	0.14 ± 0.003 a	0.49 ± 0.05 b
	STAC4.1	9.18 ± 0.04 a	0.84 ± 0.002 b	2.83 ± 0.078 b	3.36	0.07 ± 0.003 b	1.01 ± 0.20 a
	STAC10	9.29 ± 0.03 a	0.99 ± 0.029 a	2.98 ± 0.085 b	3.01	0.06 ± 0.004 c	1.22 ± 0.19 a
	STAC42.1	8.89 ± 0.11 a	0.36 ± 0.028 c	1.66 ± 0.031 c	4.61	0.07 ± 0.003 b	1.19 ± 0.18 a
Scotta 2	STAC 4	9.15 ± 0.14 a	1.07 ± 0.024 a	3.58 ± 0.263 a	3.35	0.06 ± 0.005 c	1.24 ± 0.07 a
	STAC4.1	8.11 ± 0.14 a	0.99 ± 0.010 b	3.12 ± 0.048 b	3.15	0.14 ± 0.001 b	1.31 ± 0.18 a
	STAC10	9.12 ± 0.18 a	1.04 ± 0.003 a	3.44 ± 0.053 ab	3.31	0.16 ± 0.006 a	1.30 ± 0.10 a
	STAC42.1	8.57 ± 0.10 a	0.62 ± 0.000 c	2.3 ± 0.000 c	3.71	0.03 ± 0.005 d	1.29 ± 0.12 a

Results are mean ± std of two technical replicates of two biological replicates. Different letters in the same column indicate values significantly different (*p* < 0.05). Eq., Equivalents.

## 4. Discussion

The production of innovative healthy, safe, and sustainable novel food and feed is a “leitmotiv” within the scientific community and the agri-food sector (Willett et al., 2019), and the search for multidisciplinary approaches that optimize their production, according to a circular economy strategy, is now compulsory (Gonçalves and Maximo, 2022). Here, with the aim of evaluating the possibility to utilize PABs for the valorization of scotta, 47 dairy and ruminal isolates ascribed to *Propionibacterium* spp. and *Acidipropionibacterium* spp. were first characterized for antibiotic resistance and growth kinetics on lactose containing medium and then screened for functional and biotechnological properties prior to being evaluated for the production of bioactive molecules in scotta.

Antibiotic resistance is an undesirable trait that needs to be thoroughly assessed in food-related bacteria to limit the spreading of antimicrobial resistance genes to intestinal microbiota (Altieri, 2016). Few papers report on intrinsic or “natural” resistance of dairy PABs to several antibiotics, among which sulphonamides, nalidixic acid, oxacillin, aminoglycosides (streptomycin, kanamycin, and gentamycin), 1st and 2nd generation quinolones, polypeptides, colistin, metronidazole, fosfomycin and semisynthetic penicillin (i.e., amoxicillin) (Cummins and Johnson, 1992; Meile et al., 2008; Suomalainen et al., 2008). These resistances do not appear to be encoded by plasmids or other mobile genetic elements (Altieri, 2016; Deptula et al., 2017), although a unique strain of *P. freudenreichii* carrying three genes coding for putative antibiotic resistance-related proteins within a genomic island flanked by mobile genetic elements was found (Deptula et al., 2017). On the other hand, propionibacteria are susceptible to most β-lactams (penicillin G and A, ampicillin, cefalosporins) as well as to cyclins (tetracycline) (Suomalainen et al., 2008; Darilmaz and Beyatli, 2012; Yuksekdag et al., 2014; Campaniello et al., 2015). Here, susceptibility to ampicillin was confirmed for dairy and rumen PABs isolates. Moreover, all the isolates proved susceptible to gentamycin and streptomycin and most of them could be considered susceptible to chloramphenicol and clindamycin as reported by Campaniello et al. (2015). Macrolides showed different effects, with PABs isolates being generally susceptible to vancomycin and tetracycline but not to erythromycin. Erythromycin resistance has already been reported for *P. acnes* where it may be due to a mutation on the 23S ribosomal RNA (Ross et al., 2001). Similarly, in *P. freudenreichii* T82 strain, mutations at G2294A and G2295A in the 23S rRNA are responsible for resistance to macrolide antibiotics (Piwowarek et al., 2021). Resistance to ciprofloxacin, here confirmed in 48% of the isolates, is generally due to mutations in the QRDR region (Quinolone Resistance Determining Region) of gyrA subunit of DNA gyrase (Spencer and Panda, 2023) but the molecular basis for this phenotype should be elucidated in propionibacteria. Amoxicillin resistance is in line with propionibacteria higher resistance to semisynthetic penicillins as compared to penicillin G (Stackebrandt et al., 2006). So far, no guidelines for the evaluation of propionibacteria antimicrobial susceptibility have been released. Most of the information available for this taxonomic group derive from studies on *P. acnes* (Nord and Oprica, 2006) and no clinical breakpoints for food-related propionibacteria are registered in EUCAST or CLSI. Thus, although a meaningful analysis of propionibacteria antibiotic susceptibility will be possible solely following the release of the clinical breakpoints for these bacteria, the results here reported may contribute to elucidate the distribution and extent of antibiotic resistance in this group of food-related bacteria.

Lactose utilization in *P. freudenreichii* is conferred by a genomic island that harbors the genes encoding a sodium galactoside symporter, a β-galactosidase and an UDP glucose-4-epimerase on a mobile element (Loux et al., 2015). Accordingly, the lactose negative phenotype correlates with the absence of this genomic island (de Freitas et al., 2015). While Loux et al. (2015) reported that lactose is degraded in a binary mode (yes or no without any difference in color intensity during phenotyping step), here different degrees of API50CH acidification were observed. Further characterization of PABs isolates growth kinetics on YELactose highlighted significant differences in terms of carrying capacity, lag phase duration and growth rate. Growth rate varied between 0.018 ± 0.003 and 0.616 ± 0.0044 h^−1^ with an average of 0.134 h^−1^, higher than that reported by other authors on lactose containing medium (0.08 ± 0.02 h^−1^) (Sabater et al., 2019).

*In vivo* experiments indicated that propionibacteria probiotic potential depends mainly on the release of beneficial metabolites including acetate, propionate (Cousin et al., 2011) and bifidogenic factors (Isawa et al., 2002) that enhance human gut immunity. Moreover, in compliance with their ecology, propionibacteria generally display *in vitro* high tolerance to simulated human upper gastrointestinal tract conditions as compared to other probiotics. *P. freudenreichii* bile salts stress tolerance seems to be mediated by the induction of a general stress-response that also include the induction of superoxide dismutase and cysteine synthase (Leverrier et al., 2003). Accordingly, the twenty-five PABs isolates proved resistant to concentrations of unconjugated bile salts that fit with their survival in the gut (Mallory et al., 1973; Northfield and McColl, 1973). On the contrary, most of the isolates showed more limited survival to 1 h treatment at pH 2. Since acid stress tolerance is an indispensable feature for probiotics survival in the gastro-intestinal tract and to enhance the biosynthesis of organic acids (Guan and Liu, 2020), solely STAC 4, STAC 4.1, STAC 10 and STAC 42.1 that passed this screening test, were further characterized for osmostress resistance and biofilm formation. Salinity is about ∼0.9% in gut contents (Fordtran et al., 1965), although variations in water content of the large intestine may cause osmotic fluctuations, and higher salt concentrations may occur in the lateral intercellular spaces and crypts (Chatton and Spring, 1995; Spring, 1998). Interestingly, although with significant differences (*p* < 0.05), the four isolates proved resistant to NaCl concentrations that were largely higher than that found in the gut. Propionibacteria are also known to adhere to intestinal cells (Zárate et al., 2016) and to counteract invasive pathogens such as *Escherichia coli*, *Pseudomonas aeruginosa, Staphylococcus aureus*, and *Salmonella enterica* through competitive adhesion or co-aggregation mechanisms (Vesterlund, 2006; Darilmaz and Beyatli, 2012; Hajfarajollah et al., 2014; Nair and Kollanoor-Johny, 2017; Barzegari et al., 2020). In accordance with the observation that biofilm formation may be triggered by suboptimal or stressful conditions (Cavero-Olguin et al., 2019), a moderate production of biofilm was observed in STAC 4.1 at the highest NaCl concentration. However, these results were obtained in polystyrene microplates treated for tissue culture. Thus, a different behavior could be expected in untreated microplates that, according to Guyomarc'h et al. (2020), may boost biofilm formation due to the establishment of hydrophobic bonds between polystyrene and cell surface components.

Santivarangkna et al. (2007) reported that lyophilization affects cell viability through the induction of proteins denaturation and inactivation and cell membrane and DNA damage. Interestingly, the four isolates proved resistant to lyophilization with an average of 8.5 Log mL^−1^ viable cells in rehydrated lyophilized biomass and residual viability of 88%, higher than that found by other authors in YEL medium (<50%) (Gaucher et al., 2020). Thus, while Gaucher et al. (2019) managed to increase survival to lyophilization by triggering the induction of a stress response in *P. freudenreichii* cells, here, viable lyophilized preparations of the four isolates were easily obtained with no additional treatments.

Propionic acid is a known inhibitor of fungal growth, widely utilized as preservative by the agrifood industry. While it is mainly obtained through the petrochemical route (Unigunde et al., 2023), its production from agrifood by-products represents a more sustainable and environmentally friendly processes. Accordingly, *P. freudenreichii* fermentates containing, among the others, propionic and acetic acids and bacteriocins, find application as natural shelf-life extenders with the commercial names MicrogardTM (Du pont Danisco) and Inhibit 3600 DairyTM (Mezzoni Foods). When inoculated in scotta STAC 4, STAC 4.1, STAC 10 and STAC 42.1 produced propionic and acetic acids in a strain- and substrate-dependent fashion. Since propionic acid biosynthesis is NADH consuming and propionibacteria modulate the production of propionic and acetic acids to maintain their redox balance (Turgay et al., 2022), the observed increase in P/A ratio could be an indication of NAD/NADH imbalance, thus highlighting the need to optimize the composition of scotta to gather higher and balanced concentrations of organic acids.

Industrial applications of *P. freudenreichii* also include the production of group B vitamins (Rabah et al., 2017). These, besides occurring in dark green leafy vegetables, beans, peas and nuts (vitamin B9), and foods of animal origin (vitamin B12) (Watanabe and Bito, 2018; Singh, 2022), are also synthetized by probiotic bacteria and the possibility to obtain them by fermentation is gaining increasing importance, also due to the reduction of the consumption of animal-origin foods (Poore and Nemecek, 2018). Accordingly, *P. freudenreichii* was utilized to obtain functional sweet whey enriched in group B vitamins (Huang et al., 2019). Here, STAC 4, STAC 4.1, STAC 10 and STAC 42.1 proved capable of producing vitamins B9 and B12 metabolites, although with clear semi-quantitative differences on the two scotta samples. Therefore, looking at both the folates distribution and the annotation of some key metabolites (such as S-Adenosyl-Methionine, the cofactors NAD, NADH, NADP, NADPH and several nucleotides from guanosine and adenosine; Supplementary Table 4) associated to the metabolic profile of *P. freudenreichii*, a certain interplay between the folate cycle and methylation cycle can be hypothesized, being this latter widely described to contribute to adenosylcobalamin metabolism (Piwowarek et al., 2018; Liu et al., 2021). In particular, it is known that, in *P. freudenreichii*, adenosylcobalamin is coproduced together with the main product propionic acid (Liu et al., 2021), as confirmed in this work as well (Table 8). Thus, on the one side, the biosynthetic pathways for these two vitamins can be considered active in the four isolates, although further studies are needed to discover the potential metabolic regulation mechanisms, which would help remove the bottlenecks of vitamin B12 production in *Propionibacterium*. On the other side, scotta composition, that is strictly dependent on ricotta cheese yield and the technological conditions employed during ricotta cheese making (Cabizza et al., 2021), greatly affects vitamins production. Interestingly, the amount of vitamins B9 and B12 metabolites produced in scotta 2, with no precursor addition, by the three isolates in pure culture, were the same order of magnitude of that reported by Hugenschmidt et al. (2011) when co-culturing *Lactobacillus plantarum* and *P. freudenreichii* on sweet whey permeate. Indeed, when grown in YELactose plus lactate the selected PABs isolates showed significant increases in carrying capacity. Thus, the impact of scotta co-inoculation of selected dairy PABs with lactate producing lactobacilli on the production of vitamins and shelf-life extenders deserves to be further explored.

In conclusion, 47 PABs isolates were molecularly identified, characterized for antibiotic susceptibility and lactose utilization, and selected based on unconjugated bile salts tolerance and acids stress resistance. Four *P. freudenreichii* harboring resistance to bile salts, acid stress, osmostress and lyophilization were inoculated in scotta. On this substrate, the four isolates reached cell densities ranging from 8.11 ± 0.14 to 9.45 ± 0.06 Log CFU mL^−1^ and proved capable of producing different vitamin B9 vitamers. In addition, the four isolates showed a total production of cobalamin-derivatives (vitamin B12) in the range 0.49–1.31 mg L^−1^ thus supporting the full activity of the corresponding biosynthetic pathways, likely involving a complex interplay between folate cycle and methylation cycle required in vitamin B12 biosynthesis. These isolates appear interesting candidates for further investigation regarding the production of pro-bioactive scotta.

## Data availability statement

The original data presented in the study, and included in the Supplementary Material, are deposited in Mendeley Data and publicly available at: https://data.mendeley.com/datasets/zf337gz8pr/1 (V1) and https://data.mendeley.com/datasets/zf337gz8pr/2 (V2).

## Author contributions

FF and IM contributed to conception and design of the study. RC and FF carried out investigation. GLP, AG, GR, and ML performed the chemical analyses. GZ performed the statistical analysis. IM wrote the first draft of the manuscript and acquired the financial support. All authors contributed to manuscript revision, read, and approved the submitted version.

## Funding

This research was partially supported by the Agritech National Research Center and received funding from the European Union Next-GenerationEU (Piano Nazionale di Ripresa e Resilienza (PNRR) – Missione 4 Componente 2, Investimento 1.4 – D.D. 1032 17/06/2022, CN00000022). This manuscript reflects only the authors’ views and opinions, neither the European Union nor the European Commission can be considered responsible for them. RC scholarship was funded by Fondazione di Sardegna (Proscotta-2022 PI IM).

## Conflict of interest

The authors declare that the research was conducted in the absence of any commercial or financial relationships that could be construed as a potential conflict of interest.

## Publisher’s note

All claims expressed in this article are solely those of the authors and do not necessarily represent those of their affiliated organizations, or those of the publisher, the editors and the reviewers. Any product that may be evaluated in this article, or claim that may be made by its manufacturer, is not guaranteed or endorsed by the publisher.

## Footnotes

^1^ https://blast.ncbi.nlm.nih.gov/Blast.cgi/

^2^www.foodb.ca; last accessed: June 2023.

^3^ https://www.MetaboAnalyst.ca/
