# Supplementary material for: Propionibacteria as promising tools for the production of pro-bioactive scotta: a proof-of-concept study
Source: Front Microbiol. 2023 Jul 31;14:1223741. doi: 10.3389/fmicb.2023.1223741 (PMC10425813; doi:10.3389/fmicb.2023.1223741)

Supplementary Material

**Propionibacteria as promising tools for the production of pro-bioactive scotta: a proof-of-concept study**

Roberta Coronas^1^, Giacomo Zara^1^, Antonio Gallo^2^, Gabriele Rocchetti^2^, Marco Lapris^2^, Giacomo Luigi Petretto^1^, Severino Zara^1^, Francesco Fancello^1,ᵻ^ & Ilaria Mannazzu^1,ᵻ^

* Correspondence:

Ilaria Mannazzu, imannazzu@uniss.it; Francesco Fancello, fancello@uniss.it

Supplementary Table 1. MIC 50 and MIC 90 of *P. freudereichii* and *Acidipropionibacterium* spp. isolates

| Antibiotic | MIC50 | MIC90 | MIC50 | MIC90 |
| --- | --- | --- | --- | --- |
| (mg L^-1^) | *P. freudereichii* | | *Acidipropionibacterium spp.* | |
| AM | 0.5 | 1 | 1 | 2 |
| AMX | >128 | >128 | >128 | >128 |
| CHL | 2 | 4 | 2 | 2 |
| CIP | 2 | 4 | 1 | 4 |
| CC | 0.0625 | 0.125 | 0.0625 | 0.25 |
| ERY | 0.25 | 4 | 0.0625 | 2 |
| GENT | 2 | 8 | 1 | 8 |
| KAN | 32 | 256 | 8 | 128 |
| SPE | 256 | 256 | 8 | 256 |
| STR | 4 | 32 | 1 | 16 |
| TET | 0.5 | 4 | 2 | 4 |
| T/S | 0,125/2,375 | 2/38 | 0.0625/1.1875 | 2/38 |
| VAN | 1 | 2 | 0.5 | 1 |

Supplementary Table 2. Clinical break points reported in Methods for Antimicrobial Susceptibility Testing of Anaerobic Bacteria; Approved Standard- Seventh Edition.

| Antibiotic | CLSI resistance break point  (mg L^-1^) | EFSA ECOFF value  (mg L^-1^) |
| --- | --- | --- |
| AM |  | 2 |
| CHL |  | 2 |
| CIP | ≥4** |  |
| CC |  | 0.25 |
| ERY |  | 0.5 |
| GENT |  | 64 |
| KAN |  | 64 |
| SPE |  |  |
| STR |  | 64 |
| TET |  | 2 |
| T/S | ≥ 4/76* |  |
| VAN |  | 4 |

No clinical breakpoints for food-related propionibacteria are registered in EUCAST or CLSI. * CLSI break point for *Corynebacterium* spp. and *Lactococcus* spp..; ** CLSI break point for Gram positive Anaerobes; ^a^ ECOFF for *Staphylococcus aureus*.

Supplementary Table 3. Prediction ability and Log_2_Fold-Change (FC) variations of the different vitamin B9 vitamers (folates) when considering both fermented scotta 1 and scotta 2 samples.

VIP = Variables Importance in Projection.

| **Scotta 1** | **VIP score** | **Log_2_ FC**  **STAC 4 *vs***  **not fermented** | **Log_2_ FC**  **STAC 10 *vs***  **not fermented** | **Log_2_ FC**  **STAC 4.1 *vs***  **not fermented** | **Log_2_ FC**  **STAC 42.1 *vs***  **not fermented** |
| --- | --- | --- | --- | --- | --- |
| Folic acid | 0.85 ± 0.38 | -1.51 | -2.08 | -1.17 | -1.34 |
| Tetrahydrofolate (THF) | 0.91 ±0.58 | 0.55 | 2.79 | 0.90 | 2.75 |
| 10-Formyl-THF | 1.16 ± 0.82 | 1.88 | -3.98 | -3.91 | -3.92 |
| 5,10-Methenyl-THF | 1.19 ± 0.30 | -3.28 | -3.44 | -3.38 | -3.39 |
| 5,10-Methylene-THF | < 0.8 | 1.51 | 0.66 | 1.16 | 0.89 |
| 5-Methyl-THF | 1.55 ± 0.56 | 1.57 | 2.95 | -2.34 | -2.35 |
|  |  |  |  |  |  |
| **Scotta 2** | **VIP score** | **Log_2_ FC**  **STAC 4 *vs***  **not fermented** | **Log_2_ FC**  **STAC 10 *vs***  **not fermented** | **Log_2_ FC**  **STAC 4.1 *vs***  **not fermented** | **Log_2_ FC**  **STAC 42.1 *vs***  **not fermented** |
| Folic acid | < 0.8 | 1.54 | 1.04 | 1.64 | 0.62 |
| Tetrahydrofolate (THF) | < 0.8 | 0.92 | 1.87 | 0.79 | 1.36 |
| 10-Formyl-THF | 1.03 ± 0.33 | 2.13 | 2.16 | 0.98 | 1.33 |
| 5,10-Methylene-THF | < 0.8 | 0.75 | 1.42 | 0.83 | ns |
| 5-Methyl-THF | 1.22 ± 1.06 | 0.82 | 0.79 | 0.46 | -1.21 |
| Dihydrofolic acid | 1.33 ± 0.34 | -2.33 | -0.83 | -2.14 | -4.94 |

Supplementary Table 4. Metabolomic dataset resulting from the untargeted UHPLC-HRMS analysis containing all the annotated mass features ad key putatively identified metabolites, together with their identification score, relative abundance value, and composite isotopic mass spectrum. The supplementary table 4 is publicly available as Mendeley Data on the reserved doi: 10.17632/zf337gz8pr.1


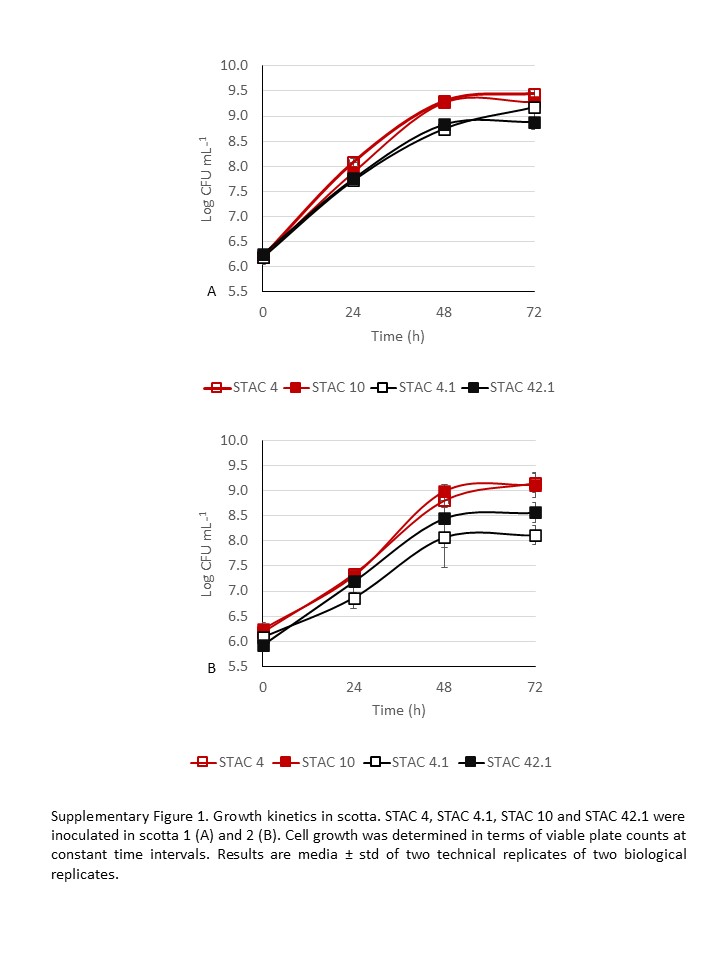

Supplement: Supplementary file 1 [file Data_Sheet_1.docx]
